# Exercise efficiency of adolescents with idiopathic scoliosis in and without Cheneau brace

**DOI:** 10.1186/1748-7161-8-S2-O58

**Published:** 2013-09-18

**Authors:** Jacek Durmala, Joanna Pajak

**Affiliations:** 1 Department of Rehabilitation, Medical University of Silesia, Poland

## Background

Untreated idiopathic scoliosis (IS) may lead to large deformations of the trunk, limiting capacity and biomechanics functions of the chest and exercise capacity, which is closely related to changes in the functioning of the cardio-pulmonary system. The conservative treatment of IS is applied in a brace treatment and various methods of physiotherapy. However, the usage of the brace may additionally decrease the cardio-pulmonary efficiency, regardless of what the beneficial effects of persistent brace therapy might be. 

## Purpose

The goal of this study was to observe whether a Cheneau brace (ChB) affects the exercise capacity (ExC) among the patients with IS based on the analysis of the two forms of exercise: Cycle Ergometer Test (CET) and the 6-Minute Walk Test (6MWT). Additional goals were to determine the relationship between factors such as curvature size (Cobb), the deformation extent and the degree of a direct effect of the ChB on ExC. 

## Methods

Fifty-Six patients (10-18 years) with diagnosed IS, who passed through the CET and the 6MWT. Tests were performed twice: once in a brace and once without it. Design: A randomized, controlled trial. 

## Results

The study showed a disability of the exercise test parameters on the CET during the test with a ChB compared to the test without a ChB. The search for a correlation between the degree and location of the scoliosis, the thoracic kyphosis degree, children’s age and the CET parameters exercise test failed in most cases. The significantly shorter average distance of 6MWT was founded in the children’s group wearing a brace compared to the group without the brace. 

## Conclusions and discussion

A ChB significantly limits the ExC. The impairment of this capacity has been demonstrated in both tests for CET and 6MWT. Both exercise tests had limited utility in assessing the negative effect of the degree and location of scoliosis on some parameters in these trials. More useful in this aspect was a 6MWT, in which was found a significant inverse correlation between the thoracic kyphosis degree and the difference in march distance with and without a brace. 
